# Roles of non-receptor tyrosine kinases in pathogenesis and treatment of depression

**DOI:** 10.31083/j.jin2101025

**Published:** 2022-01-28

**Authors:** John Q. Wang, Justin D. Derges, Alaya Bodepudi, Nikhila Pokala, Li-Min Mao

**Affiliations:** 1 Department of Biomedical Sciences, School of Medicine, University of Missouri-Kansas City, Kansas, MO 64108, USA; 2 Department of Anesthesiology, School of Medicine, University of Missouri-Kansas City, Kansas, MO 64108, USA

**Keywords:** Tyrosine kinase, Src, Fyn, JAK, FAK, PYK2, Depression, Antidepressant

## Abstract

Major depressive disorder is a chronic psychiatric disease with a high prevalence. Brain mechanisms for depression at cellular and molecular levels are far from clear. Increasing evidence from clinical and preclinical studies reveals critical roles of the non-receptor tyrosine kinase (nRTK) superfamily in the pathophysiology, symptomatology, and therapy of depression. To date, several nRTK members from three nRTK subfamilies, i.e., the Src family kinase (SFK), the Janus tyrosine kinase (JAK) and the focal adhesion kinase (FAK) subfamilies, may connect to the intracellular, intranuclear, and synaptic signaling network linking chronic stress to depressi- and anxiety-like behavior. These SFK/JAK/FAK nRTKs are abundantly expressed in the prefrontal cortex and hippocampus, two core limbic regions implicated in depression, and are enriched at synaptic sites. In various acute or chronic animal models of depression, the nRTKs were significantly altered (up- or downregulated) in their phosphorylation, expression, subcellular/subsynaptic distribution, and/or function. Stress that precipitates depressive behavior also influenced the interaction of nRTKs with other signaling molecules and downstream substrates, including ionotropic and metabotropic glutamate receptors. The commonly-used antidepressants showed the ability to alter nRTK activity. In sum, the limbic SFK/JAK/FAK nRTKs are sensitive to stress and undergo drastic adaptations in response to chronic depression. These long-lasting adaptations contribute to the remodeling of signaling network or synaptic plasticity critical for the vulnerability to depression and the therapeutic efficacy of antidepressants.

## 1. Introduction

A tyrosine kinase is a protein enzyme that transfers a phosphoryl group from a nucleoside triphosphate donor, e.g., ATP, to the specific amino acid tyrosine on a protein, i.e., a posttranslational process called phosphorylation. By this liable and reversible process, tyrosine kinases control the expression, subcellular distribution, protein-protein interactions, and function of modified proteins. Tyrosine kinases represent a large family, including receptor tyrosine kinases and non-receptor tyrosine kinases (nRTK). The former are cell surface receptors for growth factors, cytokines, and hormones, which transmit ligand-mediated extracellular signals to the cytoplasm and nucleus. The latter are cytosolic enzymes linking to and thus regulating multiple signal transduction cascades. Most nRTKs are subjected to autophosphorylation or phosphorylation by different nRTKs at tyrosine sites in the activation loop, leading to an increase in enzymatic activity [1]. In the brain, many nRTK members are abundantly expressed in neurons or glial cells. Since some nRTKs are enriched at synaptic sites and are robustly autophosphorylated in response to changing synaptic input, these nRTKs are involved in synaptic plasticity in relation to the strength and efficacy of synaptic transmission or susceptibility of a variety of neurological and neuropsychiatric disorders [2, 3].

Major depressive disorder is a neuropsychiatric disorder that negatively affects millions of people. As a mental illness with high prevalence and high rate of treatment-resistance and recurrence, its cause and risk factors have been investigated extensively in preclinical and clinical studies. However, despite all efforts, brain mechanisms underlying depression risk are still obscure. Increasing evidence indicates that chronic exposure to stress seems to be effective in precipitating depression-like behavior in experimental animals. Such long-term incubation of stress is thought to induce neuroadaptations of distinctive signaling pathways in neurons and/or glial cells of brain regions implicated in depression. These adaptive changes then contribute to enduring depressive behavior, although precise molecular mechanisms are far from clear.

nRTKs may connect to the signaling network critical for linking chronic stress to depression-like behavior. It is noted that several subfamilies of nRTKs, including the Src family kinase (SFK), the Janus tyrosine kinase (JAK) and the focal adhesion kinase (FAK), are expressed in the brain at a high level [4, 5]. Emerging evidence shows that nRTK members from these three families are sensitive to stress that precipitates depression. In fact, in different animal models of depression, acute or chronic stress altered autophosphorylation, expression, subcellular/subsynaptic distribution, and function of these nRTKs in the limbic forebrain regions critical for depression, such as the prefrontal cortex (PFC) and hippocampus. These plastic changes were either pathogenic in promoting the vulnerability for depression or compensatory in normalizing behavioral responses to stress, depending on signaling pathways that nRTKs interact with, downstream substrates of nRTKs, brain regions and circuits, models of depression, etc. Standard antidepressants can also affect the nRTK pathway to exert their therapeutic effects. This review discusses the brain nRTK’s sensitivity to depression and potential roles of nRTKs in the pathophysiology and symptomatology of depression with a focus on recent progress. In addition, the potential antidepressant properties of nRTKs and associated signaling pathways are analyzed.

## 2. SFK family

Brain SFKs may be sensitive to stress and may undergo long-term adaptive changes during the development of depression. As a core intracellular signaling pathway, SFKs interact with a large number of substrates, including receptor, signaling, structural, and enzymatic proteins. By interacting with different substrates, SFKs either affect the susceptibility to depression or serve as a dynamic element in a molecular mechanism underlying the effect of antidepressants. Among SFK members enriched in the brain (Src, Fyn, Yes, Lyn, and Lck), Src and Fyn are particularly interesting as they are abundant at synaptic sites and have been demonstrated to have a role in depression. One study found that depressive stress altered autophosphorylation of SFKs in mice, which served as an inducible element to reduce the vulnerability to depression [6]. In details, this study showed that forced swim induced an increase in mouse passive behavior (immobility time), indicating a state of despair or depression. Notably, forced swim also induced the autophosphorylation of SFKs at a conserved pan-Y416 site (leading to activation of SFKs) in the hippocampus (Table 1 Ref. [6-17]). Two key SFK members, Src and Fyn, were affected in this event as autophosphorylation was elevated in both Src and Fyn proteins immuno-precipitated from the hippocampus. Active Src/Fyn then tyrosine-phosphorylated an Ig-superfamily and synapse-rich protein, signal regulatory protein *α* (SIRP*α*; also known as SHPS-1 and p84), and increased the binding of SIRP*α* to its downstream protein, tyrosine protein phosphatase Shp2. This Src/Fyn-SIRP*α*-Shp2 pathway seems to be mobilized to reduce depression risk since mutant mice lacking most of the tyrosine-phosphorylated cytoplasmic region of SIRP*α* or lacking the SIRP*α* ligand CD47 manifested the increased immobility time relative to wild-type (WT) mice. Moreover, the tricyclic antidepressants imipramine and desipramine induced tyrosine phosphorylation of SIRP*α* in the hippocampus of WT mice.

G_*αs*_-coupled 5-hydroxytryptamine 6 (5-HT_6_) receptors may be another target of SFKs. An early study showed that Fyn directly bound to the intracellular C terminus of 5-HT_6_ receptors [18]. This direct protein-protein interaction enabled 5-HT_6_ receptors to enhance autophosphorylation of Fyn, which thereby activated the mitogen-activated protein kinase (MAPK)/extracellular signal-regulated kinase 1/2 (ERK1/2). The role of Fyn in linking 5-HT_6_ receptors to ERK1/2 is noteworthy as the ERK signaling was significantly downregulated in the PFC and hippocampus of depressed humans and animals and various antidepressants acted in part through normalizing the downregulated ERK activity [19]. Reciprocally, the Fyn binding increased 5-HT_6_ receptor activity. Given that 5-HT_6_ agonists exhibit antidepressant properties [20] and 5-HT_6_ receptors have a high affinity for a set of antidepressants [21], Fyn by interacting with 5-HT_6_ receptors could serve as a signaling mechanism for antidepressant effects.

Fyn may interact with neurotrophic factors for the etiology and treatment of depression. Brain-derived neurotrophic factor (BDNF) and its receptor, i.e., the tropomyosin receptor kinase B (TrkB) receptor, were lowered in their expression or phosphorylation in the PFC and/or hippocampus of depressed humans and animals, and BDNF has been generally shown to have antidepressant properties [19, 22, 23]. A recent study provided evidence supporting that BDNF exerts its antidepressant effects partially via a signaling mechanism involving Fyn. According to Diniz *et al*. [24], blockade of the angiotensin II receptor 1 (AGTR1) with losartan shifted angiotensin II to interact with AGTR2 receptors. Activated AGTR2 then recruited Fyn to TrkB receptors, leading to transactivation of TrkB receptors. Through the linkage of AGTR2 to TrkB by Fyn, the AGTR1 blocker reduced the immobility time in the forced swim test and is thus considered to have therapeutic potential as a novel class of antidepressants. In addition to BDNF, glial cell line-derived neurotrophic factor (GDNF) is regulated by an SFK-dependent pathway. The antidepressant amitriptyline increased SFK phosphorylation in rat C6 astroglial cells [7]. Amitriptyline also induced GDNF production, which was blocked by SFK inhibitors. Since astrocytes are implicated in processing the effect of antidepressants by producing neurotrophic/growth factors [7, 22], SFKs could contribute to the antidepressant efficacy by mediating the neurotrophic factor production in astrocytes.

Postpartum depression affects approximately 15% of mothers after childbirth [25]. In ovariectomized female rats that were treated with pregnancy-associated hormones (estrogen and progesterone) to create a hormone-stimulated pregnancy, abrupt withdrawal of hormone treatment precipitated depression-like behavior, mimicking the postpartum depression in humans [26]. In a similar model of postpartum depression in mice, Src phosphorylation was reduced in the hippocampus [8]. The reduced Src activity, as an initial step, led to a series of subsequent events, i.e., decreased tyrosine phosphorylation of *N*-methyl-D-aspartate (NMDA) receptors (see below), impaired neurogenesis in the hippocampus, and finally depression-like behavior. Consistent with this scenario, systemic administration of the Src inhibitor dasatinib caused depression- and anxiety-like behaviors in hormone-stimulated pregnancy rats [8].

Of note, at present, there remain significant limitations in our ability to investigate molecular mechanisms underlying pathophysiology of human mental illnesses due to the difficulties of convincingly modeling human brain disorders in animals. Major depression is among disorders whose clinical features in humans are difficult to model in animals [27]. Depression includes various sets of symptoms, and a major subset of symptoms that can be measured objectively in rodents include anhedonia, psychomotor behavior, and homeostatic symptoms. Acute stress paradigms such as the forced swim test involve short-term stress applied to normal rodents, which is different from the chronically developed human depression pathology. Moreover, the forced swim test measures the altered behavioral responses to stress, which include but are not limited to depression. Thus, this test may not provide definitive evidence for a depression phenotype [27-29]. Nevertheless, despite the limitations, assays based on acute stress exposure might be useful in initial screens [27]. Future studies will aim to (1) generate useful rodent and invertebrate models with the strong construct (or etio-logic), face, and predictive (or pharmacological) validity, and (2) utilize chronic stress models more frequently and broaden the scope of behavioral assays. These endeavors are deemed helpful for promoting analysis of roles of nRTKs in pathogenesis of depression and antidepressant action.

## 3. SFK-glutamate receptor interactions in depression

**Fig. 1.**
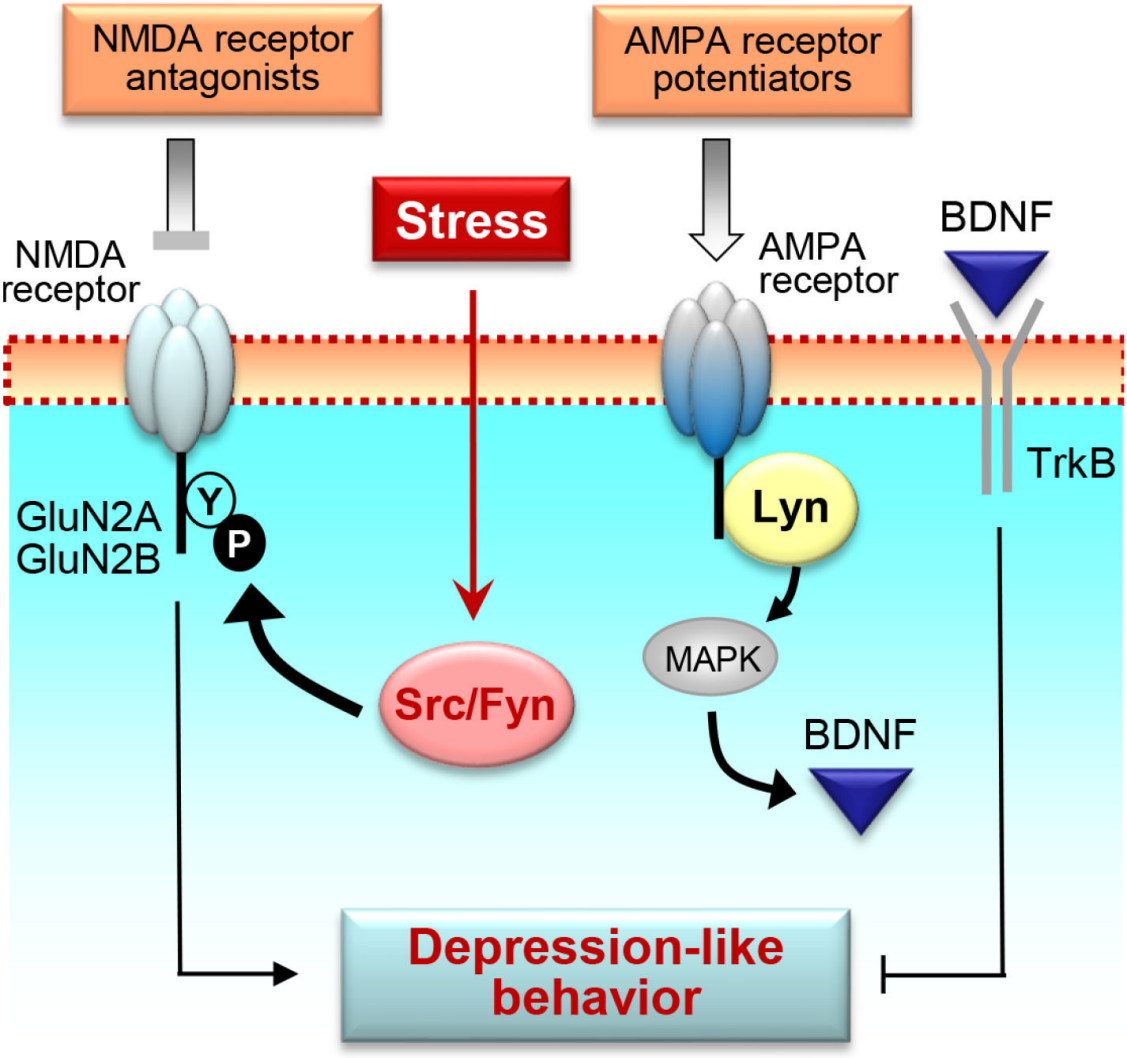
A schematic diagram illustrating roles of SFKs in interacting with ionotropic glutamate receptors in relation to depression and antidepressant action. Chronic stress that causes depression-like behavior could enhance autophosphorylation (activation) of synapse-enriched SFKs (mainly Src and Fyn) in the brain regions implicated in the pathogenesis of depression, including the prefrontal cortex and hippocampus. Active Src/Fyn then phosphorylate NMDA receptor GluN2A/2B subunits at a specific set of tyrosine residues, which enhances NMDA receptor activity and promotes the vulnerability to depression. On the other hand, AMPA receptors upon activation could activate a neuron-enriched SFK member Lyn. Active Lyn in turn causes translocation of cytosolic MAPKs to the nucleus to upregulate BDNF gene expression. Enhanced BDNF-TrkB signaling could then participate in molecular events critical for alleviating depression-like behavior. As such, NMDA receptor antagonists and AMPA receptor potentiators are generally considered to possess the antidepressant value and may produce their therapeutic effects partially via a signaling mechanism involving SFKs.

Glutamatergic transmission is a non-monoamine-based system implicated in depression and antidepressant action [30]. Since SFKs vigorously phosphorylate NMDA receptors in response to changing synaptic input [31, 32], SFKs could modulate NMDA receptors to influence the state of depression and the effect of antidepressants. Functional NMDA receptors are assembled by obligatory GluN1 (formerly known as NR1) and modulatory GluN2 subunits, mainly GluN2A (NR2A) and GluN2B (NR2B) [33]. The GluN2A C-terminal region harbors multiple tyrosine sites phosphorylated by Src and/or Fyn, including Y842, Y1292, Y1325, and Y1387 [34-36]. Among these sites, Y1325 seems to be particularly significant. Phosphorylation of Y1325 by Src potentiated NMDA receptor channels in medium spiny neurons of the mouse striatum [36]. Forced swim activated SFKs and phosphorylated GluN2A at Y1325 in striatal neurons (Fig. 1). Mutation of Y1325 to phenylalanine (Y1325F) prevented the phosphorylation at this site in transgenic mice *in vivo* and induced antidepressant-like behavior (less immobile). Thus, the SFK-GluN2A coupling links swim stress to behavioral immobility.

In addition to GluN2A, GluN2B is a substrate of SFKs and is implicated in depression (Fig. 1). In mice, forced swim induced parallel increases in phosphorylation of SFKs and GluN2B Y1472 in the mouse hippocampus [6]. This increase in GluN2B Y1472 phosphorylation was absent in Fyn-deficient mice. Thus, Fyn mediated the upregulation of GluN2B Y1472 phosphorylation and this Fyn-GluN2B pathway is considered to be a metabasis processing NMDA receptor plasticity and behavioral immobility in response to forced swim. These findings are consistent with a general view that NMDA receptor antagonism is of an antidepressant value [37, 38]. However, in a different model of depression, the SFK-GluN2B pathway seems to play a different role. In ovariectomized mice, estrogen hormone treatment increased Src and GluN2B phosphorylation in the hippocampus, while withdrawal of hormone treatment induced depression- and anxiety-like behavior and reduced hippocampal Src and GluN2B phosphorylation [8]. Repeated systemic administration of NMDA prevented depression-like behavior, while the GluN2B inhibitor Ro25-6981 or Src inhibitor dasatinib precipitated depression behavior in ovariectomized mice treated with hormones.

The *α*-amino-3-hydroxy-5-methylisoxazole-4-propionic acid (AMPA) receptor was found to signal through SFKs. The SFK member Lyn was physically associated with AMPA receptors in the cerebellum, a brain area implicated in depression [39], and was rapidly activated after stimulation of the receptor [40] (Fig. 1). Active Lyn then recruited MAPKs to increase BDNF expression. This Lyn-MAPK-BDNF pathway is intriguing as it may contribute to the antidepressant efficacy of AMPA receptor potentiators in various animal models of depression [37, 38, 41].

Other glutamate receptors which SFKs interact with are metabotropic glutamate (mGlu) receptors. Among three functional groups of mGlu receptors, group I mGlu receptors (mGlu1/5) have been most extensively studied for their linkage to depression and the antidepressant property [2]. Group I mGlu receptors are coupled to G*α*q proteins and activation of them activates phospholipase C*β*1, which in turn hydrolyzes phosphoinositide into inositol-1,4,5-triphosphate (IP_3_) and diacylglycerol (DAG). Through IP_3_ and DAG, mGlu1/5 receptors actively modulate various cellular and synaptic events [42]. It has been found that mGlu5 receptors in the rat brain were tyrosine-phosphorylated [43]. Recently, mGlu5 receptors were found to be sensitive to depression as this receptor underwent adaptive upregulation in its expression in the adult rat striatum following prolonged social isolation [44]. This upregulation appears to be partially mediated by a signaling mechanism involving SFKs based on the findings that (1) the SFK member Fyn interacted with striatal mGlu5 receptors, (2) this Fyn-mGlu5 interaction was increased following social isolation, and (3) the SFK inhibitor PP2 reduced the upregulation of mGlu5 expression in the striatum of socially isolated rats [9]. Of note, antidepressant activity has been seen following the use of antagonists and negative allosteric modulators selective for mGlu5 receptors [45, 46].

In summary, SFKs exhibit the ability to interact with ionotropic glutamate receptors. By influencing NMDA and AMPA receptors, SFKs play a role in the antidepressant property of NMDA receptor antagonists and AMPA receptor potentiators. In addition, SFKs were recently found to interact with mGlu receptors. By interacting with mGlu5 receptors, Fyn facilitates the upregulation of striatal mGlu5 expression in socially isolated rats showing depression-like behavior. It is possible that the Fyn-mGlu5 interaction serves as an essential element in a molecular mechanism linking social isolation to depressive behavior.

## 4. JAK family

The JAK family of nRTKs contains four members: JAK1, JAK2, JAK3, and tyrosine kinase 2 (TYK2). The canonical JAK signaling cascade is initiated by ligand-mediated activation of a gp130 receptor subunit, which promotes autophosphorylation of JAKs. Active JAKs induce phosphorylation of transcription factors known as signal transducers and activators of transcription (STAT). As a key pleiotropic intracellular cascade expressed in the mammalian brain, JAKs and STATs link various signals from cytokines and growth factors to nuclear gene expression and to multiple types of synaptic plasticity. The JAK/STAT pathway is known to play significant roles in immune and inflammatory responses. Increasing evidence shows that neuroinflammation is involved in the pathogenesis of depression, and the JAK/STAT pathway may be related to the efficacy of antidepressants [47]. Indeed, several studies show that the JAK2 signaling is critical for antidepressants. Saad *et al*. [10] found that the antidepressant venlafaxine after chronic administration markedly decreased immobility time in depressed female rats following ovariectomy. In parallel, venlafaxine enhanced phosphorylation of JAK2, STAT5, and ERK1/2 and expression of BDNF in the hippocampus. Thus, venlafaxine may exert its antidepressant effect partially though activating the JAK2/STAT5 pathway. The JAK2 signaling may also contribute to the efficacy of ketamine, a rapid-acting antidepressant. Patton *et al*. [11] reported that a single injection of ketamine rescued cognitive impairments in depression induced by chronic intermittent cold (CIC) in rats. CIC stress reduced JAK2 phosphorylation in the orbitofrontal cortex, which was reversed by ketamine. The JAK2 inhibitor AG490 prevented the ketamine-induced beneficial behavioral effect and reduction of local field potentials in the orbitofrontal cortex evoked by stimulation of excitatory thalamic afferents. In cortical neuronal cultures, JAK2 inhibition prevented the ketamine-stimulated expression of the neural plasticity-related protein Arc. In addition, inhibiting interleukin-6 or its downstream JAK/STAT pathway in the orbitofrontal cortex impaired rat cognitive flexibility [48]. These results support JAK2 as a player in the signaling pathway processing the therapeutic effect of ketamine. Consistent with this, erythropoietin, a hematopoietic growth factor, seems to produce the antidepressant-like effect. The brain is among the organs where significant erythropoietin production and secretion occur. The erythropoietin receptor is expressed in multiple regions of the central nervous system, including the cortex, hypothalamus, and hippocampus [49]. The nonhematopoietic effect of erythropoietin on depression may be attributed to its binding to the erythropoietin receptor, which results in activation of the principal downstream JAK2 signaling cascade [49]. However, in another study, forced swim increased STAT3 mRNA expression and protein phosphorylation in the rat PFC and hippocampus, which was reversed by antidepressants *N*-acetylcysteine and fluoxetine [50]. Thus, normalizing upregulated STAT3 responses to stress may be involved in mediating the effect of these antidepressants.

JAK3 is also connected to the signaling network related to depression. Stress induced by high doses of glucocorticosterone triggered phosphorylation (activation) of JAK3 in the hippocampus and caused depression-like behavior in mice [12]. The JAK3 inhibitor IV after repeated systemic administration improved behavior of depressed mice, and the antidepressant amitriptyline reduced the stress-induced phosphorylation of JAK3. Similarly, lipopolysaccharide (LPS), the main component of outer membrane of gram-negative bacteria which knowingly triggers the inflammatory cascade and induces depression in murine models [51], elevated phosphorylation of JAK3 and STAT3 in the mouse hippocampus [13]. Magnesium isoglycyrrhizinate and fluoxetine with the antidepressant value reversed the responses of JAK3 and STAT3 to LPS. These data suggest that the JAK3/STAT3 pathway in the hippocampus is upregulated in response to stress. Antidepressants may produce their effects by limiting the JAK3/STAT3 signaling.

In summary, evidence is available to support the role of the JAK2 signaling in processing effects of antidepressants. Inhibition of JAK2 prevents the antidepressant action of ketamine. In addition, antidepressants are able to reverse an increase in phosphorylation of hippocampal JAK3 in response to stress.

## 5. FAK family

The FAK family comprises two homologous members, FAK (also known as protein tyrosine kinase 2, PTK2) and proline-rich tyrosine kinase 2 (Pyk2) also named PTK2B or FAK2. FAK is activated by integrin engagement or other cell surface receptors. Active FAK serves as a signaling/scaffolding integrator for forming multimolecular complexes controlling migration, synapse formation, and neurite growth throughout brain development [52, 53]. As one of the nRTKs present in the postsynaptic density (PSD) microdomain [5], FAK participates in the formation of synaptic plasticity in response to changing synaptic input in adulthood brains [52, 54]. As such, FAK is likely to orchestrate structural and functional events at synaptic sites in response to depression. Indeed, one study found that FAK mRNA expression as detected by microarray analysis was enhanced in the striatum of patients with depression, and *FAK* gene thus might be among novel biomarkers for the depression diagnosis [14]. In a study with a commonly-used antidepressant, chronic imipramine administration reduced FAK Y397 autophosphorylation in the rat PFC [15]. Electroconvulsive shock, an effective therapy for depression, also induced a rapid and transient dephosphorylation of FAK at Y397 in the rat hippocampus [16]. These results suggest that FAK is significant in the pathogenesis of depression and antidepressant action.

Like FAK, Pyk2 is expressed in neurons. In fact, its expression in the brain is most prominent [55]. Pyk2 noticeably clusters to the PSD [56, 57] and thus can vigorously regulate synaptic plasticity [56-60]. As a Ca^2+^-sensitive kinase, Pyk2 is activated by intracellular Ca^2+^ signals and achieves its full activation through phosphorylation by Src/Fyn [61]. Sheehan *et al*. [17] found that Pyk2 is highly expressed in the rat lateral septum, an area critical for depression and antidepressant drugs [62]. Acute restraint stress for one hour decreased Pyk2 Y402 and ERK phosphorylation in the rat lateral septum, where enhancing Pyk2 expression via a local virus-mediated gene transfer produced the antidepressant effect [17]. Enhanced Pyk2 Y402 phosphorylation was also seen in the rat PFC and/or hippocampus following imipramine treatment [15] or electroconvulsive shock [16]. Thus, Pyk2 seems to undergo downregulation in its activity in response to stress [63]. Antidepressant therapy is able to increase Pyk2 activity to alleviate depression-like behavior.

In different models of depression, Pyk2 activity seems to be a catalyst for depression. Knockout of Pyk2 in mice reduced depression risk in a chronic unpredictable mild stress model of depression [64]. In Pyk2 knockout mice, stress-induced changes in dendritic spine morphology and NMDA receptor phosphorylation and expression were prevented in the amygdala. In another study, chronic restraint stress (6 h a day for 21 days) that precipitated depression induced subcellular redistribution of the active form of Pyk2 (Y402-phosphorylated) in mouse hippocampal pyramidal neurons, while total Y402-phospho-Pyk2 protein levels remained unchanged [65]. This redistribution enabled Pyk2 to phosphorylate a nuclear pore complex protein, nucleoporin p62 (NUP62), at a Pyk2-dependent site (Y422) and thereby promoted ubiquitination and proteasomal degradation of NUP62. The loss of NUP62 in this way may be pathogenic in deteriorating dendritic architecture associated with depression and cognitive impairments.

Altogether, both FAK and Pyk2 are present at synaptic sites and actively regulate synaptic transmission and plasticity. Depending on the animal model of depression, phosphorylation and activity of FAK and Pyk2 in depression-related brain regions are either up- or downregulated by stress. Altered FAK and Pyk2 activity is critical for the development of depression. Antidepressants could achieve their effects partially by normalizing FAK and Pyk2 signaling pathways.

## 6. Conclusions

In this review, we summarized roles of nRTKs in depression and antidepressant action. Available data from clinical and mainly preclinical studies show that nRTKs are linked to the signaling network relevant to depression. Several nRTK members from SFK, JAK, and FAK families were altered in their expression, subcellular distribution, and function in the limbic brain regions (PFC and hippocampus) implicated in depression by acute and especially chronic stress exposure. The SFK interaction with glutamate receptors was also sensitive to stress which promoted depression risk. These plastic changes in the nRTK pathway and associated signaling molecules constitute a biochemical basis for the remodeling of synaptic transmission and plasticity critical for either the pathophysiology of major depressive disorder or a recovery process from depression. In addition, the nRTK pathway is active in processing the efficacy of various antidepressants. Through a signaling mechanism involving nRTKs, antidepressants exert their action in improving depression-like behavior.

Evidently, it is still at an infant stage for the investigation of linkage between nRTKs and depression. Future studies will aim to explore and characterize systematic responses of nRTKs to stress in broad brain regions and circuits. In these studies, new nRTK members and new substrates of nRTKs may be identified as essential elements or biochemical markers in the signaling network critical for determining the vulnerability of depression. These new elements together with nRTK members that have been demonstrated to be sensitive to depressive stress may also be the targets of antidepressants and act to mediate the effect of antidepressant drugs. Of note, given a large number of nRTKs and their interacting signaling molecules, dynamic crosstalk is expected among these signaling regulators at different levels. It is thus intriguing to explore whether and how the crosstalk occurs and operates coherently to control cellular, synaptic, circuit, and behavioral responses to stress or antidepressants. Finally, at present, clinical studies and trials that target nRTKs in terms of molecular mechanisms and treatment of depression are limited. More clinical studies are expected to be carried out following the elucidation of nRTK-dependent mechanisms underlying the etiology of depression and the efficacy of antidepressants in preclinical experiments.

## Figures and Tables

**Table 1.** Changes in nRTK activity in response to depression or antidepressants.

nRTK	Changes in phosphorylation or other events	Models of depression or antidepressants	References
SFK	Increase in Src and Fyn autophosphorylation in the mouse hippocampus	Forced swim	Ohnishi *et al*. [6]
	Decrease in Src phosphorylation in the mouse hippocampus	Postpartum depression	Zhang *et al*. [8]
	Increase in SFK phosphorylation in rat C6 astroglial cells	Antidepressant (amitriptyline)	Abe *et al*. [7]
	No change in Src/Fyn autophosphorylation, but increase in Fyn-mGlu5 interactions in the rat striatum	Prolonged social isolation	Mao *et al*. [9]
JAK	Decrease in JAK2 phosphorylation in the rat orbitofrontal cortex, which was reversed by ketamine	Chronic intermittent cold and antidepressant (ketamine)	Patton *et al*. [11]
	Increase in JAK2 phosphorylation in the rat hippocampus	Antidepressant (venlafaxine)	Saad *et al*. [10]
	Increase in JAK3 phosphorylation in the mouse hippocampus, which was reduced by amitriptyline	High dose of glucocorticosterone and antidepressant (amitriptyline)	Gulbins *et al*. [12]
	Increase in JAK3 phosphorylation in the mouse hippocampus, which was reversed by magnesium isoglycyrrhizinate and fluoxetine	Lipopolysaccharide and antidepressants (magnesium isoglycyrrhizinate and fluoxetine)	Jiang *et al*. [13]
FAK	Increase in FAK mRNA expression in the striatum	Patients with depression	Gao *et al*. [14]
	Decrease in FAK Y397 phosphorylation and increase in Pyk2 Y402 phosphorylation in the rat PFC	Antidepressant (imipramine)	Zalewska *et al*. [15]
	Decrease in FAK Y397 phosphorylation and increase in Pyk2 Y402 phosphorylation in the rat hippocampus	Electroconvulsive shock (therapy for depression)	Kang *et al*. [16]
	Decrease in Pyk2 phosphorylation in the rat lateral septum	Acute restraint stress	Sheehan *et al*. [17]

FAK, focal adhesion kinase; JAK, Janus tyrosine kinase; mGlu, metabotropic glutamate; nRTK, non-receptor tyrosine kinase; Pyk2, proline-rich tyrosine kinase 2; SFK, Src family kinase.

## Abbreviations

AGTR: angiotensin II receptor
AMPA: *α*-amino-3-hydroxy-5-methylisoxazole-4-propionic acid
BDNF: Brain-derived neurotrophic factor
CIC: chronic intermittent cold
DAG: diacylglycerol
ERK: extracellular signal-regulated kinase
FAK: focal adhesion kinase
GDNF: glial cell line-derived neurotrophic factor
5-HT_6_: 5-hydroxytryptamine 6
IP_3_: inositol-1,4,5-triphosphate
JAK: Janus tyrosine kinase
LPS: lipopolysaccharide
MAPK: mitogen-activated protein kinase
mGlu: metabotropic glutamate
NMDA: *N*-methyl-*D*-aspartate
nRTK: non-receptor tyrosine kinase
NUP62: nucleoporin p62
PFC: prefrontal cortex
PSD: postsynaptic density
PTK2: protein tyrosine kinase 2
Pyk2: proline-rich tyrosine kinase 2
SFK: Src family kinase
SIRP*α*: signal regulatory protein *α*
STAT: signal transducers and activators of transcription
TrkB: tropomyosin receptor kinase B
TYK2: tyrosine kinase 2
WT: wild-type
